# Cardiovascular, Lymphatic, and Ocular Health in Space

**DOI:** 10.3390/life12020268

**Published:** 2022-02-11

**Authors:** Victoria Ly, Suhas Rao Velichala, Alan R. Hargens

**Affiliations:** Department of Orthopaedic Surgery, UC San Diego Medical Center, University of California San Diego, San Diego, CA 92093, USA; vily@ucsd.edu (V.L.); svelicha@ucsd.edu (S.R.V.)

**Keywords:** spaceflight, microgravity, cephalad fluid shift, endothelial dysfunction, SANS, cosmic radiation, countermeasures

## Abstract

Life on Earth has evolved continuously under Earth’s 1 G force and the protection of the magnetosphere. Thus, astronauts exhibit maladaptive physiological responses during space travel. Exposure to harmful cosmic radiation and weightlessness are unique conditions to the deep-space environment responsible for several spaceflight-associated risks: visual impairment, immune dysfunction, and cancer due to cosmic radiation in astronauts. The evidence thus reviewed indicates that microgravity and cosmic radiation have deleterious effects on the cardiovascular, lymphatic, and vision systems of astronauts on long-duration space missions. The mechanisms responsible for the decline in these systems are potentially due to cytoskeletal filament rearrangement, endothelial dysfunction, and muscular atrophy. These factors may alter fluid hemodynamics within cardiovascular and lymphatic vasculatures such that greater fluid filtration causes facial and intracranial edema. Thus, microgravity induces cephalad fluid shifts contributing to spaceflight-associated neuro-ocular syndrome (SANS). Moreover, visual impairment via retinal ischemia and altered nitric oxide production may alter endothelial function. Based on rodent studies, cosmic radiation may exacerbate the effects of microgravity as observed in impaired endothelium and altered immunity. Relevant findings help understand the extent of these risks associated with spaceflight and suggest relevant countermeasures to protect astronaut health during deep-space missions.

## 1. Introduction

Long-duration spaceflight subjects astronauts to unique conditions not present on Earth, generating altered physiological responses to environmental stress. Microgravity and exposure to cosmic radiation ultimately contribute to a decline in cardiovascular, lymphatic, and ocular health systems, which may pose significant risks to astronauts both acutely and chronically. On Earth, gravity-dependent hydrostatic forces maintain body fluid equilibrium, and the magnetosphere protects organisms from harmful cosmic radiation. Weightlessness in space eliminates the typical hydrostatic pressure gradients resulting in facial edema and headward fluid shift, a possible contributor to spaceflight-associated neuro-ocular syndrome (SANS). The purpose of this review is to summarize the current understanding of physiological changes induced by space travel and to explore potential countermeasures against maladaptive responses to spaceflight.

## 2. Fluid Shifts and Venous Changes Due to Space Travel

**Figure 1:**
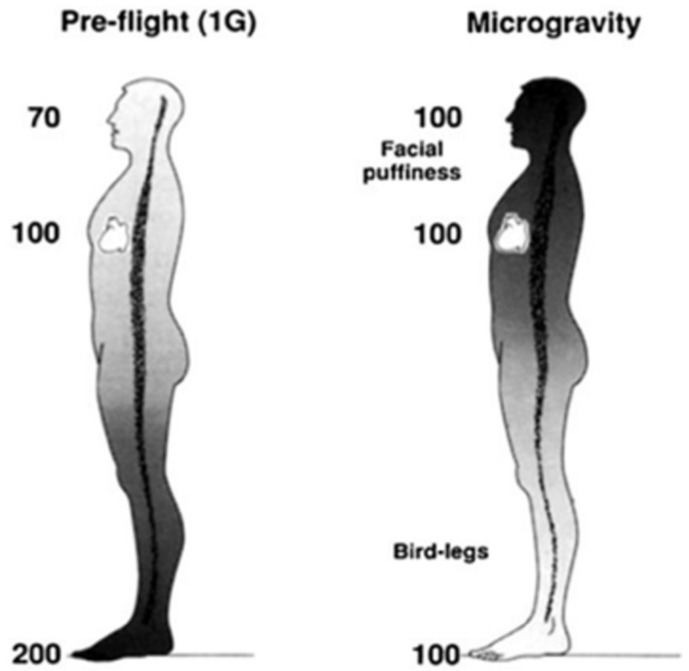
Hypothetical arterial blood pressures (mmHg) while upright in 1 G and during microgravity. Modified from Hargens and Richardson, 2009.

The loss of Earth’s gravitational pressures and forces cause many adaptations and maladaptations regarding blood circulation, vasculature, and headward fluid shifts in space. Previous studies have highlighted the physiological responses to microgravity, such as decreases in venous pressures, loss of plasma volume, and orthostatic intolerance, among other symptoms [1]. In space, the absence of blood pressure gradients that normally exist under Earth’s gravity redistributes mean arterial pressure within the head and feet (Figure 1) [1], ultimately resulting in facial edema and volume loss in the lower extremities [2]. Essentially, microgravity negates gravity-dependent hydrostatic pressures within the body, resulting in bodily fluid columns virtually disappearing and a new equilibrium state established with regards to body fluid and volume redistribution. Symptoms of space-adaptation syndrome, a type of motion sickness common among astronauts in space, may arise from microgravity-induced headward fluid shifts [3]. Headward fluid shifts and facial edema may also alter an astronaut’s perception of taste and aroma due to swelling of the paranasal sinuses, reducing food palatability and decreasing caloric intake. Consequently, body mass decrease is an indicator of cardiovascular degradation and bone and muscle loss [4,5]. Skeletal muscle cytoskeletal activity is gravity-dependent; gravitational forces impact protein mass and phenotype of slow-twitch skeletal muscle fibers. Skeletal muscle fibers, therefore, atrophy in microgravity via changes in myosin heavy chain and sarcoplasmic reticulum protein isoforms [6].

Another impact on venous adaptations in microgravity is increased coagulation in the cephalad venous system [7]. As demonstrated by measuring increases in internal jugular vein cross-sectional area, the absence of Earth’s gravity increases venous pressure and decreases cranial venous drainage. Consequences include venous distension, endothelial damage, and potential hypercoagulability [7]. Spaceflight-induced endothelial dysfunction and vascular endothelium remodeling result in cardiovascular deconditioning. Microgravity affects the migration of endothelial cells, which are vital to maintaining the structure and stability of vascular cell walls. Simulated microgravity experiments showed that vascular cells increase nitric oxide production in space, which causes actin rearrangement and induces irregular endothelial cell migration [8]. These responses are often due to cosmic radiation and, more importantly, changes in gravity, often leading to unhealthy fluid shifts and other implications. To mitigate the impact of microgravity on headward fluid shifts and maintain orthostatic tolerance and endothelial function, lower body negative pressure (LBNP) devices are sometimes available in present-day spacecraft such as the International Space Station. LBNP chambers are vacuum devices that surround one’s lower body and simulate gravitational stress to redistribute venous fluid caudally into the lower extremities [9]. 

Exposure to various forms of ionizing radiation during spaceflight also negatively affects the venous system, as the cardiovascular system may be prone to defect when exposed [10,11]. More specifically, weightlessness and cosmic radiation may impair endothelium-dependent vasodilation via cAMP-dependent hyperpolarization of vascular smooth muscle cells [12]. Microvasculature, which serves an important role in normal organ function, is also potentially impacted by space radiation [13]. Factors such as these are the focus of recent studies documenting that mortality related to cardiovascular disease is higher among lunar flight astronauts than in age-matched United States populations [14]. Thus, future studies aiming to improve the radioprotective equipment in space are needed to protect the long-term cardiovascular health of those engaging in space travel.

Potential countermeasures against physiological impacts on the cardiovascular system that are currently being considered include pharmacological methods or employing a protective water shield within the walls of the spacecraft [13,15]. Water absorbs cosmic radiation [15] and can therefore negate its effects on a spacecraft and its inhabitants. Additionally, the water within the walls can serve multiple purposes and even be drinkable. Ultimately, physiological and external countermeasures are being considered to help reduce the impact of cosmic radiation on astronauts’ cardiovascular systems, but more research must be conducted before these methods are implemented, especially for a long-duration space mission.

## 3. Microgravity Analogs on the Lymphatic System

The lymphatic system is important to help control immunity in the peripheral tissues [16,17] and the central nervous system [18]. It is responsible for preventing and resolving edema, maintaining normal tissue fluid volume [19] and immunologic responses, and clearing extravascular proteins and cells from cancers [16,17]. The lymphatic system also plays a critical role in CSF clearance from the cranial space [20], such that impaired CSF outflow into lymphatics during spaceflight may be associated with several pathological conditions, including SANS [18,20].

**Figure 2:**
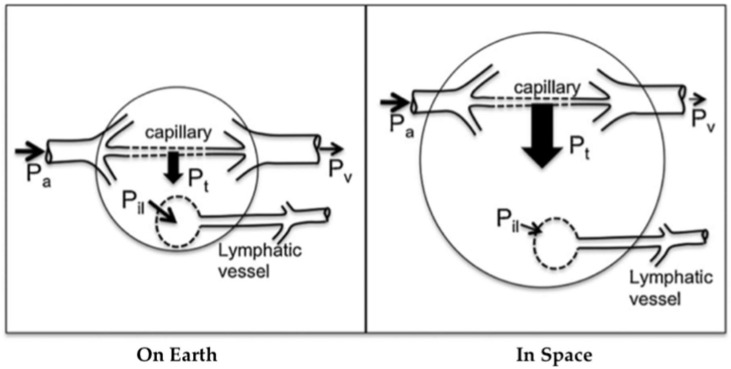
Altered capillary transmural pressure (blood to tissue) due to microgravity. The arterial pressure P_a_, venous pressure P_v_, transmural pressure P_t_, and interstitial fluid to lymph pressure gradient P_il_ are shown, with larger arrows indicating greater pressure gradients. In space, the loss of tissue weight reduces tissue hydrostatic pressure further, generating even higher transmural pressure. The increase in transmural pressure causes increased fluid flow into the tissue and, thus, edema. Because lymph flow depends highly on tissue deformation and local hydrostatic gradients, lymphatic flow may be reduced in space. Arterial flow depends on the input arterial pressure P_a_ involved (see Figure 1). Modified from Hargens and Richardson 2009.

Lymph flow depends on local tissue deformation and gravity-dependent hydrostatic gradients [21]. Intrinsic and extrinsic lymphatic pumping mechanisms transport lymph while valves prevent retrograde flow. These pumps are extremely sensitive to stretch and shear on muscular lymphatics [22] and lymphatic pressure and flow [23]. Therefore, the microgravity environment compromises lymphatic function due to several deconditioning mechanisms: loss of hydrostatic pressure, decreased sensory information, reduced mechanical stimulation, and altered Starling-Landis pressures (Figure 2) [21]. Tissue weight in microgravity is essentially zero due to the absence of gravitational acceleration, leading to decreased interstitial fluid pressure and increased transmural pressure. These changes alter Starling-Landis pressures and shift the Starling equilibrium to greater net filtration into tissues. It is hypothesized that the loss of tissue weight in conjunction with altered blood pressure dynamics described in Figure 2 increases transcapillary fluid transport toward tissues, consequently leading to issues such as facial edema [19,21]. A rat study conducted in simulated microgravity showed that a 2 week head-down tail suspension (HDT) model strongly inhibited pressure-/stretch-stimulated lymphatic pumping in cervical, thoracic, and mesenteric lymphatics. Since the lymphatic system relies on a gravity-dependent pressure gradient for lymph flow, it is highly likely that such inhibition was directly caused by altered lymph dynamics during HDT. Further, cephalad fluid shift inhibited both passive and active cervical lymphatic pumps, suggesting that facial edema during spaceflight may be related, in part, to decreased lymphatic drainage [19]. It is not clear how microgravity influences lymphatic pumping, but upper body edema may indicate reduced tissue fluid transport from tissues to circulation through the lymphatic system. Potential methods to stimulate CSF drainage during spaceflight include pneumatic compression devices [24] and manual lymphatic drainage techniques or pharmacologically stimulating lymphatic drainage from the intracranial space [20]. However, the development of these countermeasures has yet to be studied.

The lymphatic vasculature is lined by the endothelial glycocalyx (GCX) [25], a carbohydrate-rich matrix anchored to the cytoskeleton that contributes to the maintenance of body fluid compartments [26,27]. The GCX regulates fluid, solute, and macromolecule transfer from vessels into the sub-glycocalyx and interstitial spaces [28,29]. Elevated levels of atrial natriuretic peptide (ANP), known to occur during the first 24 h of spaceflight, are associated with GCX shedding, which results in diffuse vascular hyperpermeability and fluid shifts [30,31]. However, GCX shedding has not been studied under weightless conditions; therefore, it remains undetermined. Further research regarding altered GCX function in microgravity is needed.

Microgravity and cosmic radiation acutely and chronically affect the CNS and immune system in mice [32]. Space radiation and modeled microgravity have been shown to induce immune dysfunction [33] and affect the count of circulating blood cells in mice [34]. A mouse study by Mao et al. [32] indicated that mice subjected to a combination of proton irradiation and HDT showed a significantly lower lymphocyte count in the spleen compared to the control. However, the HDT condition appeared only to have mild effects on hematological assessment. Mao et al. found that proton irradiation without HDT decreased lymphocyte count in the blood by more than 50% compared to the control, and radiation-dependent differences were noted in specific lymphocyte subpopulations: reduced B-cell count and increased natural killer cell counts. These findings suggest a shift in favor of cells involved in innate immunity. Further, a reduction in leukocyte counts 30 days post-irradiation was observed, suggesting that the radiation effect is relatively long-term. It is unknown if changes in leukocyte populations will increase over time or if there is a long-term effect on immune function and homeostatic maintenance of the immune system [32]. Therefore, chronic radiation studies are needed. 

To date, most microgravity studies pertaining to the lymphatic system consist of rodent (rat and mouse) models subjected to HDT as the ground-based analog for gravitational unloading. It is important to note that these studies have limitations in human application, as the animal model and absence of actual microgravity are insufficient to understand many aspects of human physiology in space. The development of countermeasures against lymphatic and immune dysfunction in space has not yet been studied.

## 4. Ocular Health in Space

Visual impairment via spaceflight-associated neuro-ocular syndrome (SANS) is currently a high-profile risk associated with spaceflight. It is likely caused by microgravity-induced headward fluid shifts that lead to retinal endothelial dysfunction and damage. Some efforts to counteract these fluid shifts and prevent SANS include using lower body negative pressure, a promising countermeasure of which there are a variety of potential devices.

### 4.1. Spaceflight-Associated Neuro-Ocular Syndrome

Visual impairment of astronauts is a major concern with spaceflight and is related to headward fluid shift, a potential contributor to SANS. SANS is a collection of structural ophthalmologic and neurologic changes found in astronauts that are evidenced by visual impairment [9,12,35]. Many astronauts return from long-duration space missions experiencing short-term and sustained vision loss of differing degrees. Contributing factors include sex, age, a high-salt diet, and cardiovascular health [36,37,38]. Additionally, visual impairment is possibly a dose-dependent response to microgravity because the frequency of visual changes is reported higher among astronauts who endured longer duration space missions [39]. The significant risk of SANS has not yet led to permanent vision loss nor the need for medical intervention in space [40].

The etiology of SANS is unknown but is probably primarily attributed to a mild but chronic elevation of intracranial pressure (ICP) due to cephalad fluid shift in weightlessness, which has adverse impacts on the optic nerve [12,41]. Retinal ischemia is a secondary response to the cephalad venous congestion caused by headward fluid shift. Ischemia disrupts the generation of ATP, leading to the failure of energy-dependent cytoskeletal motor proteins and results in neural edema, axoplasmic flow stasis, and improper organelle distribution within the axon. Retinal ischemia and increased ICP consequently stress retinal endothelial cells, leading to leukocyte recruitment and activation of the inflammatory cascade. Eventually, the basement membrane proteins degrade, causing transcapillary and transretinal fluid shifts that contribute to neural edema [40].

The optic nerve sheath (ONS) is a compartment [42,43] that contains networks of trabeculae and septae that can impair orbital CSF flow. Thus, the elevated ICP that causes venous stasis in the head and neck leads to impaired CSF flow, which may result in increased subarachnoid pressure and ONS distension [12,44]. ONS distension, a theorized symptom of SANS, consequently gives rise to even more pathologies: globe flattening, choroidal folds, and optic nerve edema, among others. It is hypothesized that ONS distension initially stems from a protective mechanism that the body employs to help prevent the visual impairment it ultimately causes [42]. Additionally, it is speculated that visual impairment may be related to evidence that the intracanalicular and intraorbital optic nerves are independent of CSF-based pressure [39]. Cephalad venous stasis, along with other factors, may contribute to ONS compartment syndrome in the presence of increased ICP and are potentially the mechanisms behind the residual effects of SANS after astronauts return to Earth’s gravitational forces [36].

In terms of the impact of SANS among individual astronauts, pre-existing biochemical differences may play a role in predisposing some astronauts to long-term optic nerve damage. These differences include dissimilarities in the folate- and vitamin B_12_-dependent one-carbon transfer pathways, which may cause more significant ICP increases as fluids shift headward in microgravity [39,45,46]. It is hypothesized that B vitamins contribute to endothelial dysfunction in space as they have critical roles in nitric oxide synthesis and endothelial function. The disruption in nitric oxide synthesis due to spaceflight conditions may alter retinal elasticity therefore increasing susceptibility to fluid-shift-induced ophthalmic pathologies [47].

With respect to the effect of cosmic radiation on ocular health, a mouse study indicated that mice subjected to a combination of proton irradiation and HDT displayed increased retinal endothelial apoptosis, causing retinal endothelial dysfunction [32]. These processes may synergistically interact with microgravity-induced retinal endothelial damage and amplify its complications. Again, it should be noted that the animal model and absence of the actual microgravity environment is not sufficient to understand human physiologic responses to spaceflight.

### 4.2. Lower Body Negative Pressure to Prevent SANS

**Figure 3:**
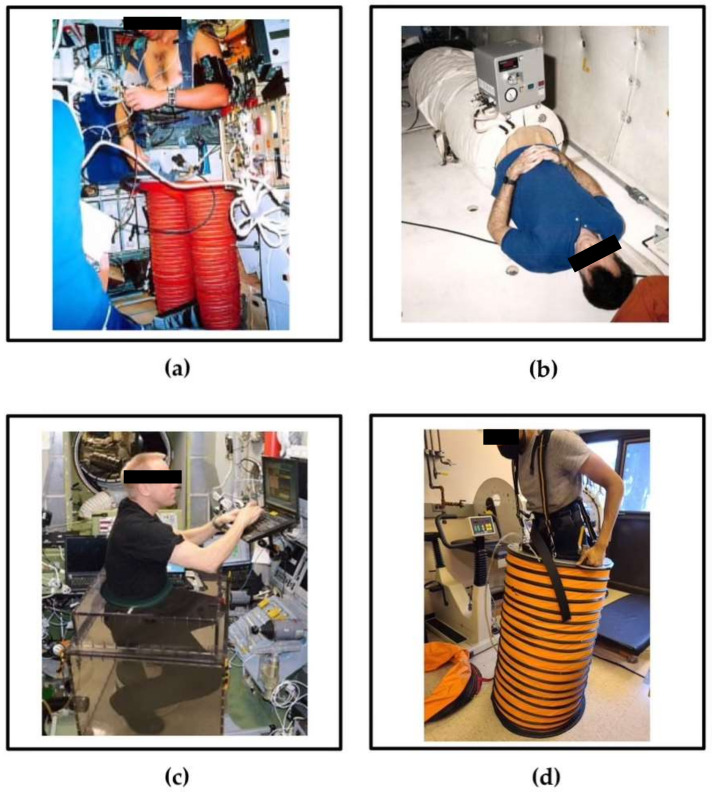
Currently used and under investigation LBNP devices: (**a**) Russian Chibis suit, (**b**) traditional static LBNP chamber, (**c**) seated LBNP device, and (**d**) self-generated LBNP device.

Issues concerning astronauts’ ocular health are currently of utmost importance as they present a major risk that currently prevents long-term space missions, such as a mission to Mars, which entails consecutive years in microgravity or 3/8 Earth gravity. Currently, lower body negative pressure devices (Figure 3) are used to prevent the causes and effects of SANS. LBNP chambers counteract headward fluid shifts by exerting static and inertial forces that simulate Earth-like gravitational conditions onto the user to potentially restore 1 G blood pressure gradients in space [39]. Preliminary evidence of the positive effects of LBNP is mainly available at 25 mmHg only, leading to questions about its efficacy for long-term spaceflights. More research is needed to truly determine long-term solutions that will alleviate the impact of SANS on astronaut visual impairment.

Astronauts are currently using and investigating a variety of LBNP devices. The Russian Chibis suit is one such device that is currently being used on the International Space Station to prepare astronauts for their return to Earth. The device is a pair of rigid LBNP trousers worn while standing that generates negative pressure using an external vacuum. The negative pressure creates a force that must be opposed by muscular contraction of the legs; therefore, the Chibis suit bears a load on the bottom of the feet and stimulates the expansion of lower body vasculature and tissue [48,49]. Another device under previous and current investigation is the traditional static LBNP chamber, which may potentially be used nightly during sleep [50]. It consists of a rigid chamber that is sealed at the level of the iliac crest around the user’s lower body while in a supine position and is connected to an external vacuum hose which generates negative pressure [51]. The Chibis suit and traditional static LBNP chamber are used in standing and supine positions, respectively, but the most common daily activity on Earth is seated posture. Sitting virtually does not occur in space, warranting current investigation of a seated LBNP device to simulate common Earth-like postural conditions during spaceflight [52]. The device consists of a rigid chamber that seals around the waist of the seated user and is attached to an external vacuum to generate negative pressure. Seated LBNP simulates gluteal, feet, and total-body load bearing to maintain musculoskeletal load and ocular health [52].

Electricity and volume are limited resources in space. A device currently under investigation that does not require either resource is the self-generated (SELF) LBNP device [53]. It consists of a collapsible chamber sealed around the user’s lower body at the level of the iliac crest and is attached to a vest. The user generates negative pressure through a continuous, squat-like dynamic motion of leg contraction followed by leg extension, in which negative pressure is generated during the extension phase due to decreased atmospheric pressure within the device. Further, the negative pressure produces increased resistance to leg extension while the vest counteracts the force that the legs generate. Therefore, the SELF LBNP device bears a musculoskeletal load on both the upper and lower body, which counteracts orthostatic intolerance. The SELF LBNP device is intended for use in standing positions in space; however, its efficacy was studied in both standing and supine positions [1,53]. The SELF LBNP device is far more efficient in power and volume than the Russian Chibis suit, traditional static LBNP chamber, and seated LBNP device as it is collapsible, has relatively low mass, and does not require an external vacuum or power source to function. an external vacuum or power source to function.

## 5. Conclusions

The evidence thus reviewed indicates that microgravity and cosmic radiation have deleterious effects on the cardiovascular, lymphatic, and vision systems of astronauts. The loss of hydrostatic pressures due to weightlessness causes fluid shifts resulting in facial edema and mild but chronically increased ICP. Thus, microgravity and cosmic radiation may combine to cause a range of maladaptive physiological responses to spaceflight. The mechanisms driving dysfunction are potentially cytoskeletal, as endothelial damage to cardiovascular and lymphatic vasculatures may cause hyperpermeability that increases capillary filtration into tissues and consequent edema. Headward fluid shifts in microgravity may be the basis for visual impairment associated with SANS, by which retinal ischemia potentially predisposes astronauts to long-term visual impairment. Harmful cosmic radiation probably exacerbates the effects of microgravity as observed in endothelial damage, altered immune cell count, and promotion of retinal endothelial apoptosis. Future studies are needed to determine the chronic effects of microgravity and cosmic radiation on the systems reviewed, especially because animal studies and the absence of actual microgravity in Earth-based models are significant limitations to understanding human physiological adaptations to the deep-space environment. Lower body negative pressure simulates Earth-like gravitational forces and is a promising countermeasure against cephalad fluid shifts and, by extension, SANS. However, further investigations of LBNP are required to determine its efficacy for long-duration spaceflight. With considerable progress made toward understanding the risks associated with space travel, future studies promise improved physiologic countermeasures to protect future space crews.
